# In-silico studies of inhibitory compounds against protease enzymes of SARS-CoV-2

**DOI:** 10.1097/MD.0000000000031318

**Published:** 2023-02-10

**Authors:** Saba Khan, Nasrullah, Abrar Hussain, Muhammad Asif, Fouzia Abdul Sattar, Fayyaz Ahmed Audhal, Muhammad Imran Qadir, Muhammad Hamid Hamdard

**Affiliations:** a Institute of Molecular Biology & Biotechnology Bahauddin Zakariya University Multan, Multan, Pakistan; b Department of Molecular Biology and Biotechnology, CASVAB, Quetta, Pakistan; c Department of Biotechnology, BUITEMS, Quetta, Pakistan; d ORIC, BUITEMS, Quetta, Balochistan, Pakistan; e Department of Chemistry, BUITEMS, QuettaPakistan; f Faculty of Biology, Kabul University, Kabul, Afghanistan.

**Keywords:** COVID-19, in silico studies, inhibitory compounds, main protease enzyme, papain like protease enzyme, SARS-CoV-2

## Abstract

In December 2019, a COVID-19 outbreak caused by SARS-CoV-2 raised worldwide health concerns. In this case, molecular docking and drug repurposing computational approaches were engaged to check the efficiency of plant-based inhibitory compounds against SARS-CoV-2 main protease enzyme and papain-like protease enzyme. Twenty phytochemical inhibitory compounds were collected. Then these compounds were screened based on Lipinski’s rule. As a result of this screening eleven compounds were further selected. Quantitative structure–activity relationships analysis was done before molecular docking to check especially the antiviral activity of inhibitory compounds. Docking validation of these compounds was checked by using online server Database of Useful Decoys: Enhanced. Binding affinity value, and pharmacokinetic properties of Aloin compound indicated that it can be used against main protease enzyme of SARS-CoV-2. So, it makes it a promising compound to follow further in cell and biochemical-based assays to explore its potential use against COVID-19.

## 1. Introduction

In China, on December 31, 2019, a cluster of pneumonia cases was reported in people concerned with the seafood wholesale market in Wuhan, Hubei Province. On January 7, 2020, it was confirmed by Chinese health authorities that this cluster is related to a novel coronavirus, 2019-nCo.^[1]^ Name as Severe acute respiratory syndrome coronavirus 2 (SARS-CoV-2) as linked with severe respiratory illness. The epidemic record shows that viruses can cause a wide range of illness from mild to severe symptoms which include death.

Coronaviruses belong to the family Coronaviridae and subfamily Coronavirinae, which are positively stranded ribonucleic acid (RNA) enveloped viruses having spikes of glycoproteins prognostic from their viral envelopes, displaying a corona or halo-like appearance.^[2]^ It was declared as a Public Health Emergency of International Concern by the World Health Organization (WHO) in January 2020 and Pandemic by WHO on March 11, 2020.^[3]^ Signs and symptoms of SARS-CoV-2 infection are fever, nasal discharge, frequent coughs, and sore throat. In case of a fatal condition, acute respiratory distress, pneumonia, and multiple organ disaster may occur.^[4]^ These symptoms are assumed to be similar to that of influenza, consisting of itching of the throat, running of nose, cough, fatigue, headache, and shortness of breath. In case of infection of COVID 19 signs persist for a longer time.^[5]^ In SARS-CoV-2 three types of proteins are coded by genome. These proteins are structural, nonstructural proteins, and accessory proteins. nonstructural proteins include Chymotrypsin like the main protease enzyme, papain-like protease and RNA-dependent polymerase. nonstructural proteins are mostly proving to be helpful in the virus life cycle. Spike (S) glycoproteins are structural proteins. These proteins play a key role in helping viral interaction with host cell receptors during its entry into the host cell.^[6]^ Moreover, for entry of coronavirus into the host cell, viral RNA must be translated by using the host cell machinery, which gives rise to virus-encoded proteins of diverse open reading frames. Open reading frames are further translated into two polyproteins that is, pp1a and pp1b. Processing of these two polyproteins gives rise to sixteen mature nonstructural proteins. Chymotrypsin like the main protease enzyme is used for the processing of thirteen nonstructural proteins. Papain-like protease enzyme is used for cleavage of three nonstructural proteins (nsp1, nsp2, and nsp3).^[7]^ Two main activities are performed by the PL-pro enzyme. These two activities are the removal of ubiquitin and ubiquitin-like protein (interferon-induced gene) IIG15 from cellular proteins. PL-pro also can inhibit the production of chemokines and cytokines. Both these chemicals are essential for activating the host immune response against infection of the virus. In this way, papain-like protease enzyme is considered a novel target to develop a drug against COVID-19 disease.^[8]^ Some antiviral drugs already used in the treatment of Middle East Respiratory Syndrome (MERS)-CoV and SARS-CoV may also be used in the treatment of SARS-CoV-2. Lopinavir, ritonavir, Remedsivir, and antimalarial drugs like chloroquine and certain drugs may also be used in the treatment of COVID 19. Remdesivir is a broad-spectrum antiviral prodrug that has antiviral in vitro activity against a set of RNA viruses such as SARS-Co-V, MERS CoV, Ebola virus, Hendra virus, and Nipah virus.^[9]^ The plant produces compounds known as phytochemical compounds, which have antimicrobial activity. Recently, a variety of inhibitory compounds are taken from plants that shows antibacterial, antifungal, and antiviral activity.^[10]^ These compounds may be flavonoids, alkaloids, flavones, phenols, and polyphenols. Phytochemical compounds have high potential and beneficial properties against viral infection and other complications related to health. These phytochemical compounds are considered as the best therapeutic agent against the coronavirus. These compounds can inhibit the replication process of the virus and also block the viral entry into host. Our study involved the screening of inhibitory compounds showing high affectivity against coronavirus protease enzyme. For screening purposes in silico study is mostly used. In silico studies are used to check that these inhibitory compounds have a strong binding affinity with the protease enzyme of SARS-CoV-2.^[11]^ To enable the rapid discovery of antiviral compounds with clinical potential, we developed an approach that combines structure-assisted drug design, computer-generated drug screening, and high-throughput screening to repurpose existing drugs by using SARS-CoV-2 main protease enzyme (3CLpro) and papain-like protease enzyme (PLpro) as target.^[12]^ Molecular docking studies are used to understand the interaction between inhibitory compounds and protein molecules. Different Autodock tools are used to check the binding mode of inhibitory compounds with the target protein.

## 2. Materials and Methods

### 2.1. Preparation of target

To perform In-silico studies of ligand-receptor interaction, 3D crystallized structure of 3CLpro protein data bank (PDB ID: 7BRO) and PLpro (PDB ID: 4RNA) were downloaded from Research Collaboratory for Structural Bioinformatics, protein data bank. Discovery studio was used to remove water and ligand molecules from the protease enzymes.

### 2.2. Selection of ligand

A Library of 20 phytochemical inhibitory compounds was made. These inhibitory compounds may be alkaloid, flavonoid, secridoids glycosides, flavanol, and phenolic compounds, etc. These compounds were then screened against the protease enzyme of SARS-CoV-2. The 3D structure of these compounds was obtained from PubChem in Simulation Description Format. List of twenty phytochemical inhibitory compounds with their pubchem compound ID number were shown in Table 1.

**Table 1:** List of phytochemical inhibitory compounds with their pubchem CIDs.

No.	Inhibitory Compounds	Type of Compound	Pubchem ID	References
1	Aloin	Phenolic	12305761	^[13]^
2	Amarogentin	Secridoids glycosides	115149	^[14]^
3	Amaroswerin	Secridoids glycosides	45359883	^[14]^
4	Apigenin	Flavonoids	5280443	^[15]^
5	Astragalin	Flavonoids	5282102	^[16]^
6	Baicalin	Flavonoids	64892	^[17]^
7	Rutin	Flavonoids	5280825	^[18]^
8	Cusparine	Alkaloids	442893	^[19]^
9	Isoquercitrin	Flavonoid glycosides	5280804	^[20]^
10	Isovitexin	Flavonoids	162350	^[21]^
11	Lignans	Phenolic	443013	^[22]^
12	Kaempferol	*Flavonoids*	5280863	^[23]^
13	Myricetin	Flavonoids	5281672	^[24]^
14	Piperitol	Phenolic	10247670	^[25]^
15	Psoralidin	Phenolic	5281806	^[25]^
16	Carpain	Alkaloid	442630	^[19]^
17	Stilbene	Phenolic	638088	^[26]^
18	Amentoflavone	Flavonoids	5281600	^[27]^
19	3-methyl Quercetin	Flavonoids	44259658	^[27]^
20	Pachypodol	Flavonoids	5281677	^[27]^

CID = compound ID number.

### 2.3. Quantitative structure-activity relationship (QSAR) analysis

The bioactivity of the eleven inhibitory compounds (table in result section) was screened using QSAR analysis using the Way2drug/PASS server (http://www.way2drug.com/PASSOnline/). Specifically, Antiviral activity of eleven inhibitory compounds and reference compound was determined by using this online software.^[28]^

### 2.4. Molecular docking

Discovery Studio was used for visualization of the 3D structure of protein and a graphic view of ligand. Modifications of protein and ligand were also achieved by using discovery studio. The modifications include the addition of polar or non-polar hydrogen atoms, removal of water molecules, and substitution of amino acids.^[29]^ Auto Dock tools 1.5.6 (https://autodock.scripps.edu/download-autodock4/) were used to explore the binding mode of inhibitory compounds onto a 3D model of protease enzyme (7BRO/4RNA) of SARS CoV 2. These Auto Dock tools were accessible from “the Scripps Research Institute” along with Auto Dock v-4.2 Programme (https://vina.scripps.edu/).^[230]^ PDB files of protease enzyme and ligand compound were opened in Autodock. Before docking, Polar-H atoms were added in the COVID-19 model (protease enzyme) followed by Kollman and Gasteiger charges calculation using the Auto-Dock tool. The macromolecule files of both protease enzymes were then saved in pdbqt format. Then inhibitory compound was selected and after some modifications, the ligand file was also saved in pdbqt format. To set the values of the grid box, option “Grid box” from “grid” was selected. Then values of the grid box were set in a very careful way. The spacing of the grid box was set up to 1.000 A (angstrom). For each inhibitory compound, dimensions (x, y, z) of the grid box were modified. Values of these dimensions (x, y, z) were set in such a way that it covers the whole targeted protease enzyme. Center grid box spacing for all three axes (x, y, z) was also set for each inhibitory compound. These pdbqt files of compound and protein were then used for running a command in Vina (https://vina.scripps.edu/). Open Source Molecular Visualization system (PyMol) software was used for the 3D visualization of ligand-protein interaction. The binding mode of a compound with amino acids residues of protein was visualized with help of PyMol.^[31]^

### 2.5. Docking validation

There are two methods to validate the docking method.

1. Re-docking

In case of presence of naturally attached ligand to protease enzyme (4RNA/7BRO) re-docking was done. Inhibitory compounds were removed from main and papain like protease enzyme and then re-docked again using Autodock tool 1.5.6. In case of re-docking co-crystalized complex was opened into a new notepad by removing heteroatoms from main protease enzyme. Same procedure was used without changing the Grid values. Binding affinity obtained by re-docking must show little deviation from actual value of co-crystalized complex.

1. Validation by Database of Useful Decoys: Enhanced (DUDE.E) database

In this case, an online automated tool (http://decoys.docking.org) was used to generate decoys of provided ligand. Decoys are compounds having similar physical properties as to the reference ligand that may not bind efficiently to the target enzyme. It was done to enhance ligand enrichment, which is crucial to assess the docking method and to exclude false positives. Almost, 102 targets were available on this online server. So properties of generated ligand decoys were then compared with these targets.^[32]^

### 2.6. Adsorption, distribution, metabolism, excretion, toxicity prediction

Pharmacodynamics properties of inhibitory compounds were analyzed by online software SwissADME (http://www.swissadme.ch). For this purpose, canonical SMILES acquired from the PubChem database (https://pubchem.ncbi.nlm.nih.gov/) were given to online software SwissADME, and results about drug-likeness and pharmacokinetic properties were calculated.^[33]^

### 2.7. Toxicity prediction

Toxicity of inhibitory compounds was analyzed by online software ToxiM (http://metagenomics.iiserb.ac.in/ToxiM) For this purpose, PDB or Simulation Description Format of desired compound or pubchem compound ID number of compound was given to the software ToxiM, and results about toxicity was determined in form of fingerprints, hybrids or descriptors.

## 3. Results

### 3.1. Quantitative structure–activity relationships (QASAR) analysis

Initially, 20 phytochemical compounds were screened. But only eleven compounds follow the Lipinski’s rule. These eleven compounds were listed in Table 2. To evaluate the bioactivity of these compounds QASAR analysis was performed. It was determined by QASAR analysis that eight compounds (Aloin, Isovitexin. Myricetin, Carpaine Kaempferol, Piperitol, Apigenin stilbene) and one reference compound show the antiviral activity. Three compounds, Cusparine, psoralidin and lignans do not show the antiviral activity as their *Pa* value is less than 0.3. While compounds Aloin, Isovitexin. Myricetin, Carpaine Kaempferol, Piperitol, Apigenin stilbene showed the antiviral activity with *Pa* value between 0.3 and 0.7. So these compounds can be used as inhibitors against protease enzyme. List of eight compounds selected by QASAR analysis was shown in Table 3.

**Table 2:** List of compounds that passed Lipinski’s rule.

No.	Compounds	SMILES	No. of Violation
1.	Aloin	C_1_ = CC_2_ = C(CO)C(=O)C_3_ = C(C_2_C_4_C(C(C(C(O_4_)CO)O)O)O)C = C(C = C_3_O)CO	1**(HD > 5**)
2.	Apigenin	C_1_ = CC(=CC = C_1_C_2_ = CC(=O)C_3_ = C(C = C(C = C_3_O_2_)O)O)O	0
3.	Carpaine	CC_1_C_2_CCC(N_1_) CCCCCCCC(=O) OC_3_CCC(CCCCCCCC(=O) O_2_) NC_3_C	0
4.	Cusparine	COC_1_ = CC(=NC_2_ = CC = CC = C_21_)CCC_3_ = CC_4_ = C(C = C_3_)OCO_4_	0
5.	Isovitexin	C_1_ = CC(=CC = C_1_C_2_ = CC(=O)C_3_ = C(O_2_)C = C(C(=C_3_O)C_4_C(C(C(C(O_4_)CO)O)O)O)O)O	0
6.	Kaempferol	C_1_ = CC(=CC = C_1_C_2_ = C(C(=O)C_3_ = C(C = C(C = C_3_O_2_)O)O)O)O	0
7.	Lignans	COC_1_ = CC(=CC(=C_1_OC)OC)C_2_C_3_C(COC_3_ = O)C(C_4_ = CC_5_ = C(C = C_24_)OCO_5_)O	0
8.	Myricetin	C_1_ = C(C = C(C(=C_1O_)O)O)C_2_ = C(C(=O)C_3_ = C(C = C(C = C_3_O_2_)O)O)O	1 (HD > 5)
9.	Piperitol	COC_1_ = C(C = CC(=C_1_)C_2_C_3_COC(C_3_CO_2_)C_4_ = CC_5_ = C(C = C_4_)OCO_5_)O	0
10	Psoralidin	CC(=CCC_1_ = CC_2_ = C(C = C_1O_)OC(=O)C_3_ = C_2O_C_4_ = C_3_C = CC(=C_4_)O)C	0
11	Stilbene	C_1_ = CC = C(C = C_1_)C = CC_2_ = CC = CC = C_2_	1 (MLOGP > 4.15)

**Table 3:** List of Compounds selected on basis of QASAR analysis (Determined by Way2drug/PASS server.

NO.	Compounds	Bioactivity (antiviral activity) *Pa* ≥ *Pi (Pa* = 0.3-0.7)
**1.**	Remdesivir **(Standard Compound**)	Greater than 0.3 (highly antiviral)
**1.**	Aloin	0.7
**2.**	Isovitexin	0.7
**3.**	Myricetin	0.5
**4.**	Stilbene	0.5
**5.**	Piperitol	0.4
**6.**	Kaempferol	0.4
**7.**	Apigenin	0.4
**8.**	Carpaine	0.4

QASAR = quantitative structure–activity relationships.

### 3.2. Docking

**Figure 1.**
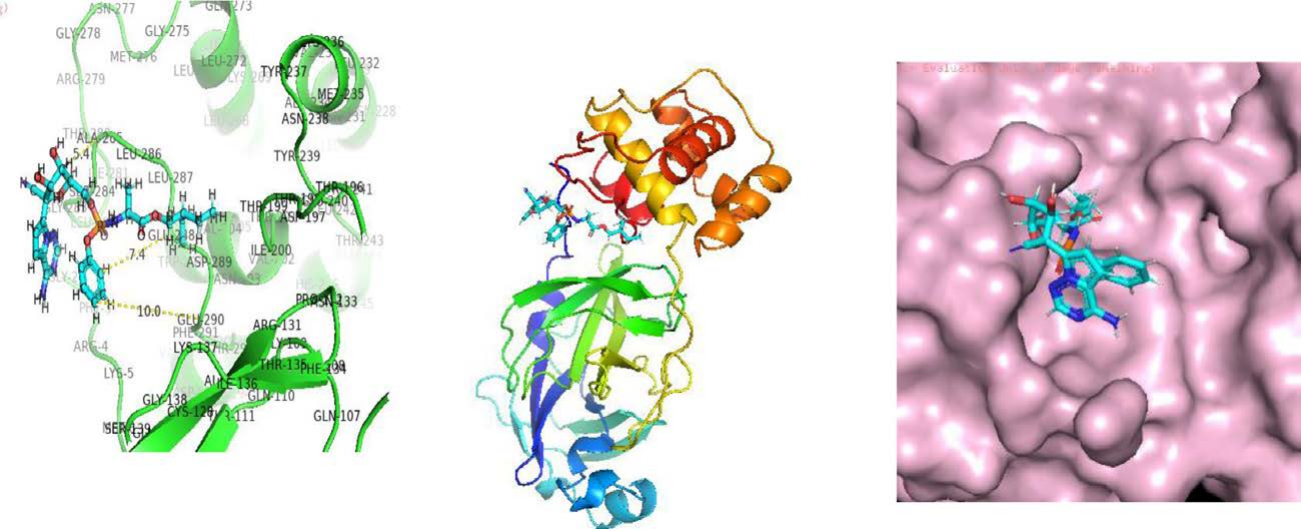
Molecular interaction of SARS-CoV 2 main protease enzyme with Remedsivir.

**Figure 2.**
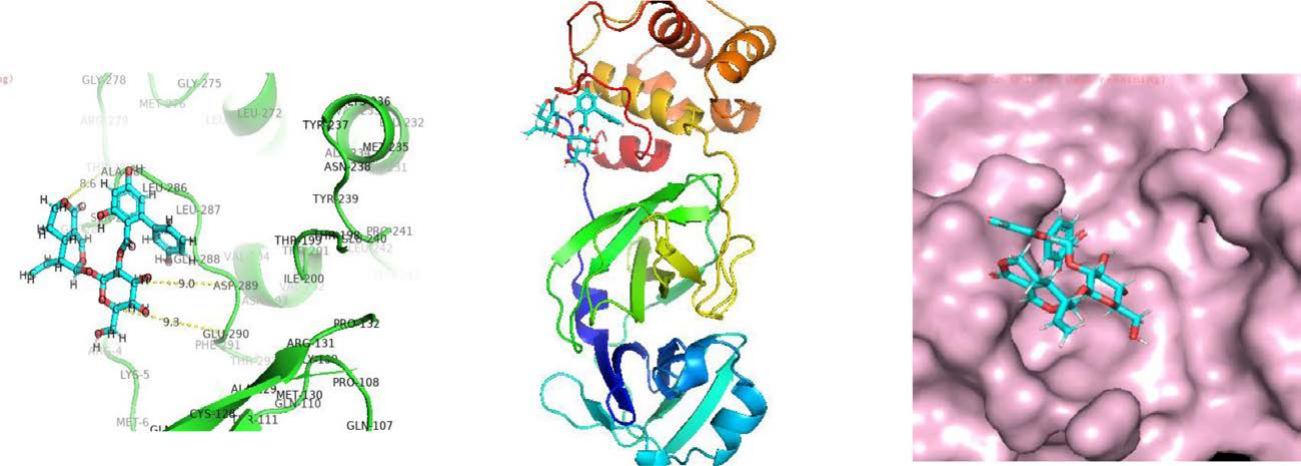
Molecular interection of SARS-CoV-2 main protease enzyme with Amarogentin.

**Figure 3.**
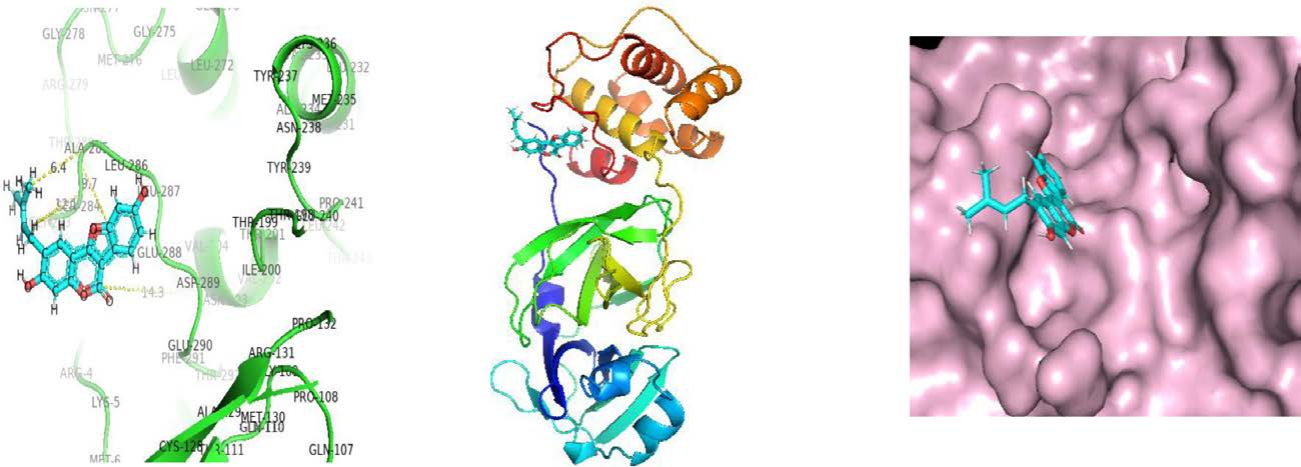
Molecular interection of SARS-CoV-2 main protease enzyme with Psoralidin. SARS-CoV-2 = Severe acute respiratory syndrome coronavirus 2.

**Figure 4.**
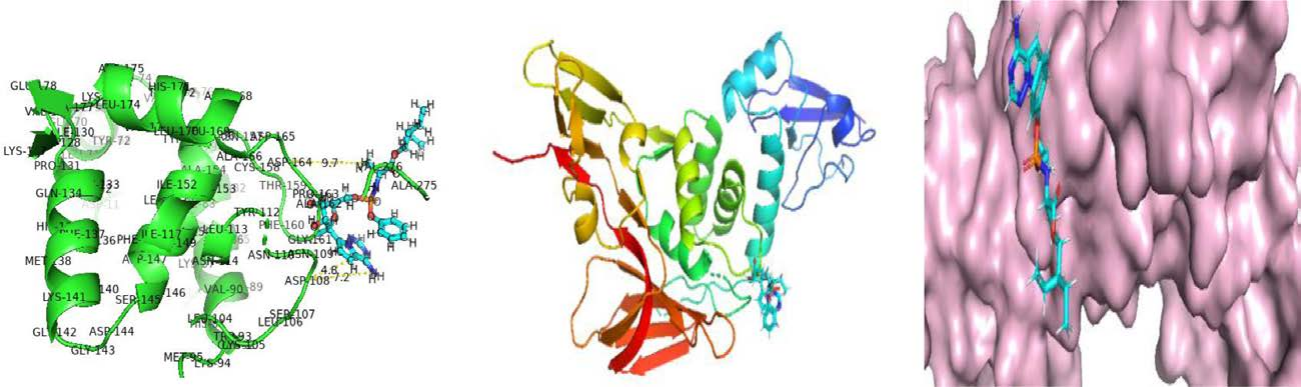
Molecular interaction of SARS-CoV-2 Papin like protease enzyme with Remedsivir. SARS-CoV-2 = Severe acute respiratory syndrome coronavirus 2.

**Figure 5.**
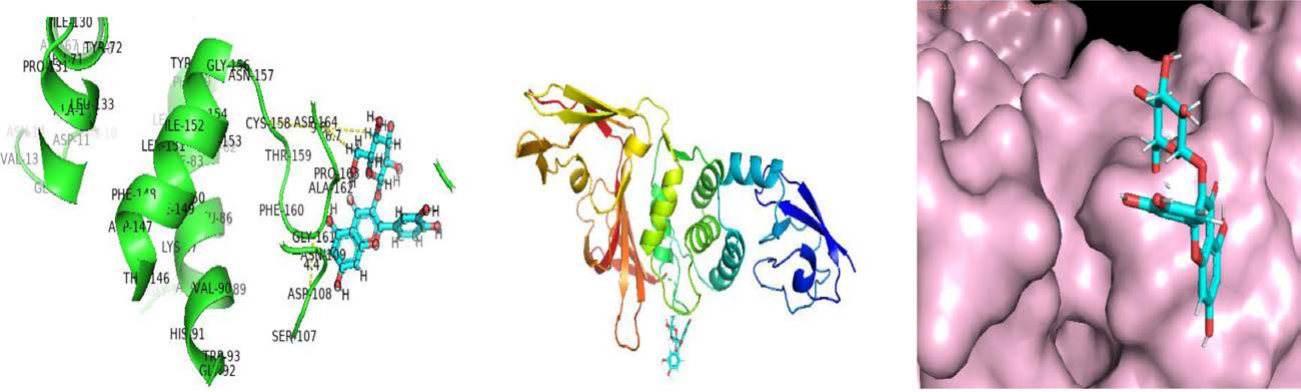
Molecular interaction of SARS-CoV-2 Papin like protease enzyme with Isoquercitrin. SARS-CoV-2 = Severe acute respiratory syndrome coronavirus 2.

**Figure 6.**
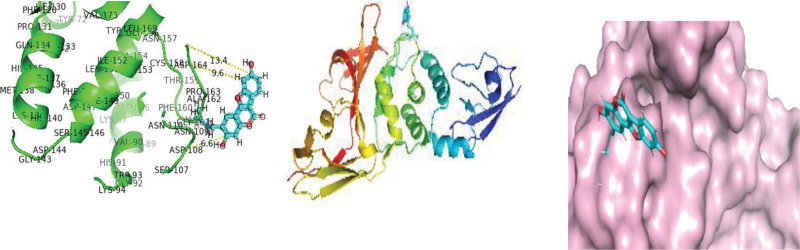
Molecular interaction of SARS-CoV-2 Papin like protease enzyme with Psoralidin. SARS-CoV-2 = Severe acute respiratory syndrome coronavirus 2.

As protease enzymes play a vital role in the replication of coronavirus. So these protease enzymes were used as a target for the discovery of drugs. In this study, different types of phytochemical inhibitory compounds were docked with the main protease enzyme and papain-like protease enzyme of SARS-CoV 2. In our study, one approved drug Remdesivir previously used in the treatment of Ebola virus now also used in the treatment of SARS-CoV-2 infection was used as a positive control for screening of antiviral phytochemical compound as a potential drug. Biovia Discovery studio (https://discover.3ds.com/discovery-studio-visualizer-download) and AUTODOCK tools were used to predict the interaction between all the listed inhibitory compounds and protease enzyme (3CLpro: PDB ID: 7BRO; PLpro: PDB ID: 4RN4 of SARS-CoV-2. In this study, ten compounds were docked with protease enzyme (7BRO/4RNA) of SARS-CoV-2 and their molecular interection was examined by an offline tool Pymol https://pymol.org/2/. (Figures 1–6). Binding affinities of selected eight antiviral medicinal plant compounds with one standard protease inhibitor (Remdesivir) were shown in Table 4.

**Table 4:**
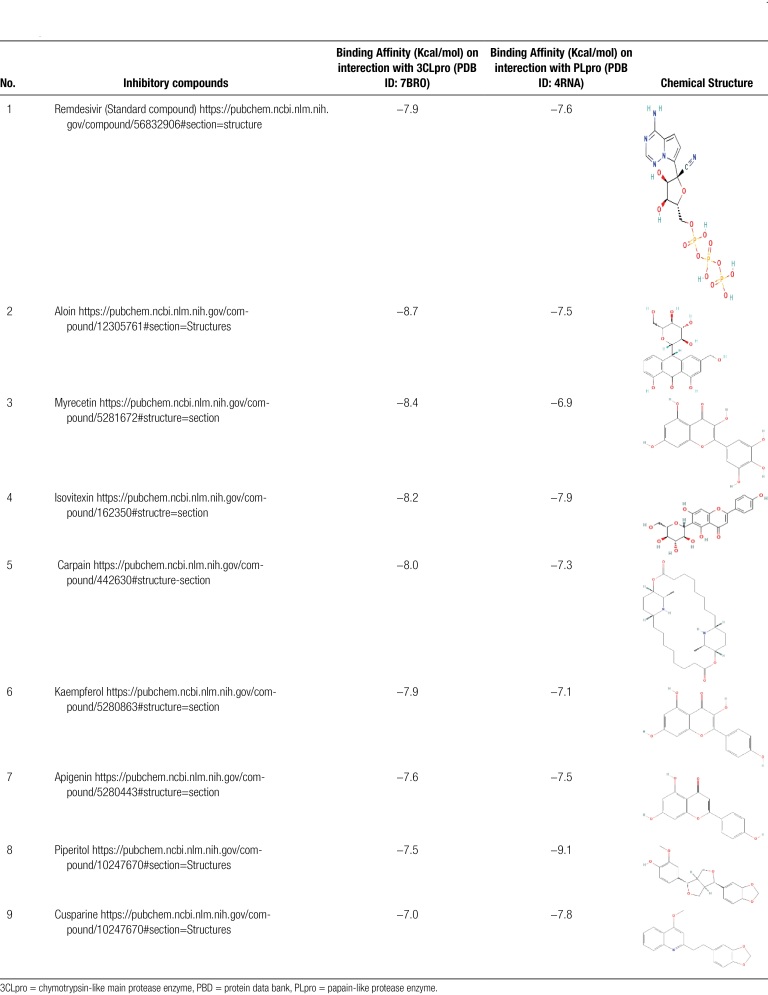
Binding Affinity of Inhibitory compounds with 3CLpro and Plpro.

### 3.3. Docking validation

Re-docking was done to check the validity of docking procedure when natural ligand is attached to protease ezyme. Aloin,myreciten compounds bind to the active site of main protease enzyme with good binding affanity value of −8.7 and −8.4 Kcal/mol when redocked. Similarly, piperitol (https://pubchem.ncbi.nlm.nih.gov/compound/Piperitol) showed the binding affinity of −9.1 when re-docked with papain like protease enzyme.

Decoys are the compounds having similar physical properties as ligand but different chemical properties. Total 102 targets are present in DUDE.E server. We have selected the fifteen protease enzyme as target. Then the decoys of aloin compound was genertaed. Decoys of this compound was then compared with targets decoys and ligands.

All targets of our ligand compounds are listed in Table 5. Gene ID, protein data bank (Pdb), matched and experimental decoys and ligands are also listed in this table. As in this study, Aloin was considered as lead compound in case of main protease enzyme target because of its high binding energy value of −8.7 Kcal/mol as compared to reference compound remdesivir. Moreover, piperitol was considered as lead compound in case of papain like protease enzyme due to its high binding energy value of −9.1 Kcal/mol. So, decoys of lead compound were generated by online tool DUDE.E to enhance the ligand enrichment. As in the original DUD, we property-matched decoys to ligands using molecular weight, estimated water-octanol partition coefficient (miLogP), rotatable bonds, hydrogen bond acceptors, and hydrogen bond donors, plus we added net charge. About 52 decoys of Aloin and 50 decoys of piperitol were generated which increase its enrichment capacity and make it possible to use against main and papin like protease enzyme respectively. Molecular weight of 418.39, miLogp value of 0.176, 3 rotatable bond, 9 hydrogen bond acceptors and 7 hydrogen bond donors of Aloin decoys were observed by DUDE.E server. Molecular weight of 356.374, miLogp value of 3.368, 3 rotatable bond, 6 hydrogen bond acceptors and 1 hydrogen bond donors of Piperitol decoys were observed by DUDE.E server. ACE target of protease class was chosen as hit for Aloin compound, as its decoys have molecular weight of 416 clustered with Aloin decoys.^[34]^

**Table 5:** Overview of representive targets (availble on online server DUDE.E).

Target class	GeneID	description	Total ligands	Clustered ligands	Experimental decoys	Matched decoys
Protease	ace	Angiotensin-converting enzyme	749	282	55	16,900
Protease	Ada17	ADAM17	1341	532	31	35,900
Protease	Bace1	Beta-secretase1	595	283	41	18,100
Protease	Casp3	Caspase-3	470	199	37	10,700
Protease	Dpp4	Dipeptidyl peptidase [9]	1939	533	167	40,950
Protease	Fa10	Coagulation factor X	3090	537	176	28,325
Protease	Fa7	Coagulation factor vii	303	114	39	6250
Protease	hivpr	Human immunodeficiency type protease	1468	536	96	35,740
Protease	Lkha4	Leukotriene 4A hydrolase	343	171	21	9450
Protease	Mmp13	Matrix metalloproteinase 13	1632	572	26	37,200
Protease	reni	Renin	391	104	46	6958
Protease	thrb	Thrombin	2109	461	255	27
Protease	Try1	Trypsin 1	924	449	117	25,980
Protease	Tryb1	Trytase beta 1	216	148	16	7650
Protease	urok	Urokinase-type plasminogen activator	372	162	44	9850

DUDE.E = Database of Useful Decoys: Enhanced.

### 3.4. Drug likeness properties analysis of inhibitory compounds

Physicochemical, pharmacokinetic properties, lipophilicity, water-solubility, pharmacokinetics, medicinal chemistry, and toxicity of nine inhibitory compounds found by docking were analyzed by SwissADME. The water solubility of these compounds revealed that Some of the inhibitory compounds were more soluble and some were moderately soluble. Other important properties like molecular weight, topological surface area (TPSA), Molecular refractivity were also studied by SwissADME. Absorption, distribution, metabolism, and excretion (ADME) properties of some inhibitory compounds were listed in Table 6:

**Table 6:** ADME analysis of phytochemical protease inhibitors by using Swiss ADME.

Parameters		PhytochemicalProtease inhibitors of SARS-CoV-2				
		Aloin	Myricetin	Isovitexin	Carpain	Piperitol
Physicochemical parameter	Formula	C12H22O9	C15H10O8	C21H20010	C21H18O11	C20H20O6
	Molecular weight	418.39 g/mol	318.24 g/mol	432.38 g/mol	478.71 g/mol	358.37 g/mol
	Value for Lipinski Rule of 5	1	1	1	0	0
	Molar refractivity	101.96	80.06	106.61	146.37	92.45
	TPSA	167.91 A	151.69 A	181.05 A	76.66 A	66.38 A
Lipophilicity	Log Po/w (iLOGP)	1.08	2.61	1.94	4.71	3.25
	Log Po/w (XLOGP3)	1.18	1.34	0.21	6.29	2.48
	Log Po/w (WLOGP)	1.69	0.17	−0.23	4.80	2.56
	Log Po/w (MLOGP)	−1.08	−0.97	−2.02	3.75	1.57
	Log Po/w (SILICOS-IT)	1.06	−0.01	0.33	3.63	2.96
	Consensus Log Po/w	−0.79	0.63	0.05	4.64	2.56
Pharmaco-kinetics	GI absorption	Low	Low	Low	High	High
	BBB permeant	No	No	No	No	Yes
	P-gp substrate	No	No	No	No	No
	Log Kp (skin permeation)	−8.94 cm/s	−7.40 cm/s	−8.79 cm/s	−4.75 cm/s	−6.71 cm/s
	CYP1A2 inhibitor	No	No	No	No	No
Water solubility	Solubility	3.14e + 01 mg/mL; 3.50e + 02 mol/L	1.18e + 01mg/mL; 1.96e-2mol/L	6.29e + 01 mg/mL; 1.46e-03 mol/L	12e-5 mg/mL; 1.70-07 mol/L	6.26e-02 mg/mL; 1.76e-04 mol/L

ADME = absorption, distribution, metabolism, and excretion, SARS-CoV-2 = severe acute respiratory syndrome coronavirus 2.

### 3.5. Toxicity prediction

For an inhibitory compound to be used as a drug, it must be less toxic and less mutagenic. So, the Toxicity of all these selected inhibitory compounds was analyzed by toxicity prediction tool for small molecules ToxiM. Some of these inhibitory compounds were less toxic and some show moderate and high level of toxicity. A Compound with classification score greater than 0.8 was considered as more toxic. Aloin compound was moderately toxic as compared to Myricetin and Isovitexin. Similarly, toxicity score value of Piperitol was less than 0.8. Toxicity score of some inhibitory compounds was listed in Table 7:

**Table 7:** Predicted toxicity score of some inhibitory compounds as evaluated by ToxiM.

Inhibitory Compounds	Predicted Toxicity Score	Category
Aloin	0.85	Moderately toxic
Myricetin	0.92	More toxic
Isovitexin	0.88	More toxic
Carpain	0.73	Less toxic
Piperitol	0.76	Less toxic

## 4. Discussion

Plants are widely used in the drug discovery process due to their massive medicinal properties. Moreover, plants are a natural source of phenols, alkaloids, flavonoids, terpenes, steroids, lignans, secoiridoid glycosides, and polyketides. Plants have anti-microbial and antiviral activities due to the presence of these bioactive compounds. Researchers are using these medicinal plants for the discovery of drugs. Since bioactive compounds obtained from plants can be targeted against a disease causing protein.^[35]^ For prediction of the binding site and drug designing in a short time, the molecular docking technique is used. To make a stable complex structure with ligand compounds, protein-ligand docking is mostly used.^[36]^ For replication of coronavirus, proteolytic processing of protease enzyme is essential. If protease enzymes are not properly working it inhibits the replication of the virus.^[37]^ Thus, a variety of bioactive compounds obtained from plants were targeted against protease enzymes of coronavirus by using in silico study. There are two types of protease enzymes, main protease enzyme, and papain-like protease enzyme.^[38]^ Remedsivir is a broad-spectrum antivirus, which has antiviral activity against a set of viruses like Nipah virus, Hendra virus, respiratory syncytial MERS, and SARS-CoV.^[9]^ In our study, twenty phytochemical compounds were screened on basis of Lipinski’s rule. Only eleven compounds follow the Lipinski’s rule. To evaluate the bioactivity of these compounds QASAR analysis was performed. It was determined by QASAR analysis that eight compounds and one reference compound show the antiviral activity. Three compounds, Cusparine, psoralidin and lignans do not show the antiviral activity. While compounds Aloin, Isovitexin. Myricetin, Carpaine Kaempferol, Piperitol, Apigenin showed the antiviral activity with *Pa* value between 0.3 and 0.7. So these compounds can be used as inhibitors against protease enzyme. So, molecular docking of these compounds was done by Autodock.

In case of docking with 3CLpro of SARS CoV-2, compounds like Aloin (−8.7 Kcal/mol), Myricetin (−8.4 Kcal/mol), Isovitexin (−8.2 Kcal/mol), Carpain (−8.0 Kcal/mol), had highly negative binding affinity values as compared to the standard inhibitory compound Remedsivir (−7.9 Kcal/mol). Moreover, in the case of docking with papain-like protease enzyme of SARS COV-2, compounds like Piperitol (−9.1 Kcal/mol), Isovitexin (−7.9), Cusparine (−7.8 Kcal/mol), had highly negative binding affinity values as compared to standard compound Remdesivir (−7.6 Kcal/mol). Validity of docking method as checked by DUDE.E online server indicated that Aloin compound had 52 decoys which increased its enrichment capacity and make it possible to use against main protease enzyme of SARS-CoV-2.

ADME properties of these compounds were determined by online software Swiss ADME. Physicochemical properties, lipophilicity, water-solubility, pharmacokinetics, drug-likeness property, and medicinal chemistry of all these ligand compounds were determined. Piperitol has high binding affinity value of 9.1 Kcal/mol against papain like protease enzyme. This compound had low toxicity and also follow the Lipinski’s rule. Similarly, Aloin compound had highly negative binding affinity value of −8.7 Kcal/mol against main protease enzyme of SARS-CoV-2. As the main protease enzyme had major rule in viral replication as compared to papain like protease enzyme. Therefore, Aloin compound may be hypothetically selected as drug against protease enzyme of SARS-CoV-2. So, this compound can be further tested by biochemical assays, as its medicinal chemistry exclaims that it has zero Pan-Assay Interference Compounds (PAINS) alert. This compound follows Lipinski’s rule of 5 and the LD50 value for this compound was found to be 500 mg/kg. So, this compound has a moderate level of toxicity and can be used against COVID-19.^[39]^

## 5. Conclusion

In this excessive health emergency that the world is fronting, a solution has to be established quickly to protect lives around the sphere. While some researchers are searching repairable molecules using synthesis techniques, another way is to encourage customary medicine for incisive lead compounds (hits) from plants. Perceptive that various phytochemical compounds are known, we can denote the bioinformatics and computational chemistry assets to help in learning diverse actions of medicinal plants. This study was directed to identify the best compound from nine phytochemicals and one artificial antiviral compound Remedsivir by use of molecular docking and Adsorption, distribution, metabolism, excretion, toxicity characteristics. The reactivity of COVID-19 main protease enzyme with eight phytochemical inhibitory compounds and indicated that a more stable complex is acquired with Aloin (−8.7 Kcal/mol), followed by Myricetin (−8.4 Kcal/mol), Isovitexin (−8.2 Kcal/mol), Carpaine (−8.0 Kcal/mol) and Kaempferol (−7.9 Kcal/mol), Similarly, the reactivity of COVID-19 papain-like protease enzyme with eight phytochemical compounds exhibited that more stable complex is acquired with Piperitol (−9.1 Kcal/mol), followed by Isovitexin (−7.9 Kcal/mol) and Cusparine (−7.8 Kcal/mol). As main protease enzyme had the major role in viral replication so the Aloin compound was considered best inhibitor against main protease enzyme due to its highly negative binding affinity value compared to reference compound. Number of decoys generated by Aloin compound also increased its enrichment capacity and made it possible to use as best inhibitor against main protease enzyme Moreover, it follows the Lipinski’s rule and other pharmacokinetic properties. This compound also had the moderate toxicity value. So, it makes it a promising compound to follow further in cell and biochemical-based assays to explore its potential use against COVID-19.

## Author contributions

All the authors contributed equally.

**Conceptualization:** Abrar Hussain.

**Formal analysis:** Saba Khan, Nas Rullah, Muhammad Asif, Fayyaz Ahmed Audhal, Muhammad Hamid Hamdard.

**Investigation:** Muhammad Asif.

**Methodology:** Saba Khan, Nas Rullah, Fouzia Abdul Sattar.

**Project administration:** Fayyaz Ahmed Audhal.

**Resources:** Muhammad Hamid Hamdard.

**Software:** Nas Rullah.

**Supervision:** Abrar Hussain, Muhammad Hamid Hamdard.

**Visualization:** Nas Rullah.

**Writing – original draft:** Abrar Hussain, Fouzia Abdul Sattar, Fayyaz Ahmed Audhal.

**Writing – review & editing:** Abrar Hussain, Fouzia Abdul Sattar, Fayyaz Ahmed Audhal.
